# Bacterial and microeukaryotic assemblage transitions during a harmful algal bloom in the Nakdong River

**DOI:** 10.1093/femsec/fiag057

**Published:** 2026-06-02

**Authors:** Dayun Kang, Min-Ji Kim, Seungjun Lee

**Affiliations:** Department of Food Science and Nutrition, Pukyong National University, Busan 48513, Republic of Korea; Department of Food Science and Nutrition, Pukyong National University, Busan 48513, Republic of Korea; Department of Applied Biosciences, Kyungpook National University, Daegu 41566, Republic of Korea

**Keywords:** Harmful algal blooms (HABs), Microcystins (MCs), Microbial community dynamics, Freshwater microbial ecology, Hydrologically regulated rivers

## Abstract

Harmful algal blooms (HABs) represent major ecological disturbances that can alter microbial community composition and reduce ecosystem resilience. Here, we investigated bacterial and microeukaryotic assemblage transitions during a bloom-affected period in the Nakdong River, a regulated river system in South Korea. By integrating microcystin (MC) measurements, cyanobacterial dominance, water-quality parameters, and amplicon-based community profiling (16S rRNA gene and ITS2), we characterized how microbial communities reorganized along gradients of bloom intensity. MC concentrations increased markedly across the sampling period, and early-phase qPCR data showed concurrent increases in *mcyE* gene abundance through 6 August 2021. Bacterial communities shifted from Actinobacteriota- and Proteobacteria-dominated assemblages toward Microcystaceae-enriched communities. Microeukaryotic communities similarly transitioned from diverse Chlorophyta, fungi, and ciliates to Chlamydomonadaceae- and ciliate-dominated assemblages under high-MC conditions. Both domains exhibited significant reductions in alpha diversity with increasing bloom intensity, although microeukaryotic responses were comparatively moderate. Correlation analyses revealed strong associations of MC concentration and *Microcystis* abundance with declining pH, elevated BOD, and the enrichment of bloom-associated taxa. These results indicate that bloom-associated conditions in this regulated river system were accompanied by parallel restructuring of bacterial and microeukaryotic assemblages. The integration of environmental and microbial indicators presented here provides a framework for understanding HAB-associated microbial reorganization and for informing monitoring strategies in hydrologically altered freshwater ecosystems.

## Teaser

Harmful algal blooms in rivers rapidly reshape microbial communities, synchronizing changes in bacteria and microscopic eukaryotes under regulated flow conditions created by river weirs.

## Introduction

Freshwater is among the planet’s scarcest and most indispensable natural resources, providing critical support for drinking water supply, agriculture, and industrial activities (Gleick and Cooley 2021, Ingrao et al. 2023). Although water covers more than 70% of the Earth’s surface, only ∼3% exists as freshwater, and less than 1% is readily accessible for human use (U.S. Bureau of Reclamation 2020). These limited resources are increasingly threatened by climate-driven changes in hydrology, irregular precipitation patterns, and anthropogenic pressures, which together degrade water quality and intensify ecological disturbances in riverine systems (Dallas and Rivers-Moore 2014, Markovic et al. 2017, Jeremias et al. 2018).

Microbial communities play fundamental roles in freshwater ecosystems by regulating nutrient cycles, degrading organic matter, and supporting food-web interactions (Berninger et al. 1993, Balser et al. 2002). Because they respond rapidly to environmental perturbations, shifts in microbial assemblages serve as sensitive indicators of ecosystem transitions (Rocca et al. 2019, Liu and Salles 2024). During harmful algal blooms (HABs), microbial interactions can influence bloom development through processes, such as nutrient regeneration, organic matter mineralization, and competition for resources (Mrdjen et al. 2018, Wilson et al. 2022). Yet bacterial and microeukaryotic communities—despite their interconnected ecological functions—are often analyzed separately, leaving key uncertainties about their coupled responses to bloom intensification.

A defining feature of HABs is the release of cyanotoxins, among which microcystins (MCs) are the most prevalent and hazardous. MCs are hepatotoxic, potentially carcinogenic, and of such concern that the World Health Organization (WHO) has established a guideline value of 1 μg/l in drinking water (World Health Organization 2020). Exposure pathways extend beyond ingestion, with recent studies demonstrating airborne transmission through aerosols and indirect exposure via contaminated irrigation water (Kim J et al. 2023). These findings emphasize that HABs represent both ecological disturbances and emerging public health risks. Understanding the ecological contexts under which blooms develop and persist is therefore fundamental to developing predictive tools and management strategies.

South Korea faces increasing challenges from HABs, particularly in the Nakdong River, one of the nation’s largest and most socially significant waterways. The river supplies drinking water to nearly one-third of the Korean population, meaning that any deterioration in water quality directly affects millions of residents. Unlike other major rivers in Korea, the Nakdong River has distinctive geomorphological characteristics that heighten HAB vulnerability. In particular, the lower 160 km of the river forms an almost flat gradient, producing exceptionally low flow velocities and naturally prolonged residence times (Academy of Korean Studies, n.d.; Ministry of Environment 2016). This intrinsic hydrological stagnation is a well-recognized feature of the system and contributes to persistent nutrient and organic-matter accumulation in the lower reach.

Hydrodynamic alterations following the construction of several large weirs have further intensified these stagnation effects by increasing water levels and extending retention time (Lee et al. 2018), thereby creating conditions that are increasingly conducive to cyanobacterial proliferation. Over the past decade, HAB duration in the Nakdong River has expanded more than 2.5-fold, with MC concentrations frequently surpassing WHO guidelines (Lee et al. 2022, Kim J et al. 2023). The detection of airborne MCs in communities adjacent to the river further highlights the multifaceted exposure risks associated with these events (Lee et al. 2019, Mo et al. 2022). These features make the Nakdong River both ecologically vulnerable and societally important, underscoring the need for an integrated analysis of HAB–environment–microbe interactions.

Despite intensive monitoring of HABs, previous research in the Nakdong River and other sites has often focused narrowly on cyanobacterial abundance or toxin concentrations. While such studies provide critical benchmarks, they rarely capture the broader restructuring of microbial ecosystems during bloom events. Both bacterial and eukaryotic communities undergo shifts that are not only taxonomic but also functional, with potential consequences for nutrient cycling and food-web stability (Woodhouse et al. 2016, Mo et al. 2022). Bacterial assemblages contribute to nutrient regeneration that sustains cyanobacterial growth (Paerl 1996, Gerphagnon et al. 2015), whereas microeukaryotic groups—including Chlorophyta, fungi, and ciliates—may compete with or indirectly facilitate bloom persistence (Paerl et al. 2001, Gopakumar et al. 2009). However, systematic evaluations of how both microbial domains respond to HAB-associated gradients remain limited.

Another key limitation is the integration of physicochemical and biological datasets. Environmental parameters such as pH, temperature, dissolved oxygen (DO), suspended solids (SS), and nutrient availability interact in complex, nonlinear ways to shape HAB dynamics (Rovelli et al. 2024). For example, reduced flow velocity and increased hydraulic retention can modify organic matter accumulation and suspended material dynamics, thereby influencing aquatic microbial and planktonic community structure (Paerl et al. 2016). Likewise, fluctuations in pH and nutrient concentrations can selectively promote or constrain particular lineages (Tripathi et al. 2018, Props and Denef 2020). Although these relationships have been documented individually, multivariate analyses linking water quality, toxin levels, and microbial diversity remain scarce.

Given these limitations, there is a clear need to examine HABs through an integrated ecological and molecular framework. Such an approach requires not only quantifying cyanotoxin levels but also linking them to shifts in both bacterial and microeukaryotic communities. This integration can reveal whether microbial groups exhibit parallel compositional transitions, whether specific taxa serve as indicators of bloom development, and how diversity is constrained under elevated MC concentrations. Importantly, these insights extend beyond descriptive ecology: clarifying microbial–environmental relationships provides a foundation for establishing early-warning indicators and improving mitigation strategies in freshwater systems.

In this study, we investigated microbial dynamics during an HAB episode in the Nakdong River by integrating environmental measurements, toxin quantification, qPCR assays, and amplicon-based community profiling. Specifically, our objectives were threefold: (i) to characterize compositional shifts in bacterial and microeukaryotic communities along gradients of MC concentration and cyanobacterial ratio; (ii) to assess changes in alpha diversity and identify discriminant taxa associated with bloom conditions; and (iii) to explore correlation networks among environmental parameters, MC concentration, cyanobacterial ratio, and microbial communities to infer ecological drivers of HAB-associated restructuring. By addressing these aims, this study advances our understanding of how HABs influence microbial ecosystem organization in a river system of national importance and underscores the value of integrated, multidomain approaches for elucidating ecological responses to bloom intensification under coupled climatic and anthropogenic pressures.

## Materials and methods

### Study area and sample collection

**Figure 1:**
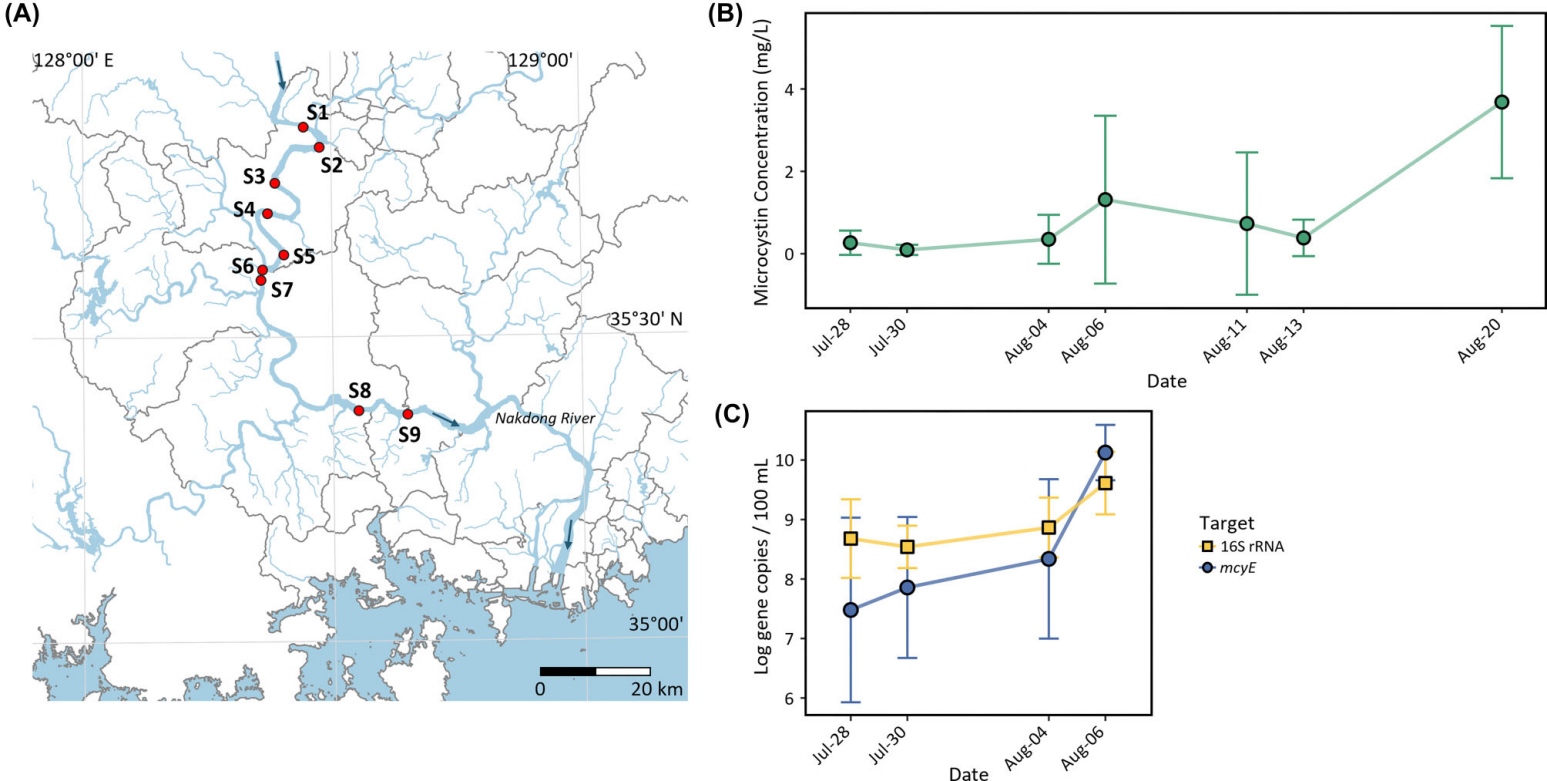
Study sites and temporal variation in MC concentrations and qPCR results. (A) Geographic distribution of the sampling sites (S1–S9) along the Nakdong River. Sampling sites are indicated, and the arrow shows the direction of river flow. (B) Temporal variation of MC concentrations (mg/l; mean ± SD) across the actual sampling dates. (C) qPCR results showing abundances of total bacteria (16S rRNA gene) and potentially toxic *Microcystis* (*mcyE*), expressed as log gene copies per 100 ml (mean ± SD). In panels B and C, the *x*-axis represents the actual sampling dates on a common temporal scale.

This study was conducted along a 94-km stretch of the Nakdong River, from Goryeong-gun (Gyeongsangbuk-do) to Haman-gun (Gyeongsangnam-do), South Korea. Nine sampling sites were selected along the river (Fig. 1, Table S1). Sampling was performed one to two times per week, depending on hydrological conditions, from 28 July to 20 August 2021. Surface water samples (approximately 500 ml each) were collected as representative near-surface water samples from the main flow region at each site, rather than from cyanobacterial scums accumulated along the riverbank, using sterile glass containers. The same samples were used for both microbial community and water quality analyses and were transported to the laboratory within 4 h under refrigerated conditions (4°C). For microbial analysis, each sample was filtered through a sterile 0.22-µm membrane filter (47 mm, Whatman, UK) using a MultiVac 610-MS-T filtration system (Rocker, Taiwan). Filters were stored at −80°C until DNA extraction.

Meteorological data, including daily precipitation and the highest and lowest temperatures, were obtained from the Korea Meteorological Administration (KMA; https://data.kma.go.kr/) (Korea Meteorological Administration n.d.). Meteorological variables were assigned using data from the nearest KMA automatic weather station (AWS) for each sampling site, selected based on geographic proximity. The matched AWS stations were as follows: S1–S2, Daegu Seo-gu (AWS 846); S3–S4, Dalseong (AWS 828); S5–S7, Cheongdeok (AWS 935); S8, Docheon (AWS 903); and S9, Gilgok (AWS 944). All selected AWS stations were located within approximately 10 km of the corresponding sampling sites. Environmental variables were matched to sampling days without temporal averaging. Sampling was occasionally not feasible due to restricted site access or adverse weather conditions, and a small number of water samples were excluded when amplification quality was insufficient for downstream molecular analyses. After these exclusions, a total of 56 samples were retained for subsequent analyses.

### Water quality and MC analysis

Water temperature was measured in situ during daytime field sampling. Physicochemical parameters—including pH, DO, biochemical oxygen demand (BOD), chemical oxygen demand (COD), SS, total phosphorus (TP), and total nitrogen (TN)—were measured from the same collected samples by the K-water Water Quality Inspection Center in accordance with the Korean Standard Methods for Examination of Water Pollution (National Institute of Environmental Research 2021). MC concentrations were determined using the Microcystins/Nodularins-ADDA ELISA kit (Abraxis, USA) following U.S. EPA Method 546 (United States Environmental Protection Agency 2020). This assay has a limit of detection (LOD) of 0.05 μg/l, a limit of quantitation (LOQ) of 0.15 μg/l, and a detection range of 0.15–5.0 μg/l (manufacturer’s manual). Samples were initially analyzed as undiluted extracts; when concentrations exceeded the assay range, aliquots of the same extract were serially diluted in 10-fold increments and reanalyzed in duplicate until a quantifiable result was obtained. Final MC concentrations were calculated by multiplying the measured concentration of the diluted sample by the corresponding dilution factor. To avoid overstating analytical precision after extensive dilution and back-calculation, values exceeding 5.0 μg/l were rounded to one decimal place, and extremely high concentrations were reported using threshold notation, with values above 5000 μg/l presented as >5000 μg/l.

### DNA extraction and sequencing

Microbial DNA was extracted from the filtered membranes using the DNeasy PowerSoil Pro Kit (Qiagen, Germany) following the manufacturer’s instructions. DNA concentrations were measured with a NanoDrop 2000 spectrophotometer (Thermo Fisher Scientific, USA) and stored at −20 °C until use. The V4 region of the 16S rRNA gene and the ITS2 region were amplified using specific primers (XT-V4-F/R and ITS1/ITS2) with TruSeq adapters (Table S2). Sequencing was performed with the MiSeq Reagent Kit v2 (Illumina, USA) using a single-end 300-cycle format. Library preparation and sequencing were conducted at the KNU NGS Core Facility (Daegu, South Korea).

### Microbial community analysis

Raw sequencing reads targeting the bacterial 16S rRNA V4 region and the eukaryotic ITS2 region were processed using QIIME2 (ver. 2023.5) (Bolyen et al. 2019). Demultiplexed single-end reads were imported with manifest files, and sequence quality profiles were inspected. Low-quality reads were filtered using the quality-score method, and amplicon sequence variants (ASVs) were inferred with the DADA2 plugin (Callahan et al. 2016). Parameters were set to trim six bases from the 5′ end and truncate reads at 300 bp for bacterial sequences, and to trim five bases and truncate at 300 bp for eukaryotic sequences. Representative sequences, feature tables, and denoising statistics were generated for each dataset.

ASVs detected fewer than two times across the entire dataset were removed. Taxonomic assignments were performed with a naïve Bayes classifier trained on the SILVA 138 database (99%, 515F–806R region) for bacteria and the UNITE database (ver. 10, 99% similarity) for eukaryotes (Quast et al. 2013, Nilsson et al. 2019). Taxa classified as mitochondria, chloroplasts, or unassigned lineages were excluded from downstream analyses. After filtering, feature tables were further curated at the sample level (minimum frequency: 2), and taxonomic classification was repeated on the filtered representative sequences. To normalize sequencing depth across samples, rarefaction was performed to the minimum read depth observed after filtering. The bacterial dataset was rarefied to 1137 reads per sample and the eukaryotic dataset to 6119 reads per sample. Rarefaction depths were determined from rarefaction curves (Fig. S1) and set to the minimum sequencing depth observed, which allowed all samples to be retained in the analysis. These depths preserved within-sample diversity patterns and did not alter between-sample ordination structure, and sensitivity analyses with higher rarefaction thresholds yielded comparable community-level trends.

The cyanobacteria ratio was defined as the relative abundance of Cyanobacteria (phylum) among total bacterial reads. Samples (*n* = 56) were categorized into three bloom-intensity groups (high, middle, and low; *n* = 19, 18, and 19, respectively) based on the distribution of cyanobacteria-ratio values, corresponding approximately to the upper quantile (>50%), interquartile range (20%–50%), and lower quantile (<20%). The terms “cyanobacteria relative abundance” and “cyanobacteria ratio” are used interchangeably throughout this study.

### Quantification of target genes

Quantitative polymerase chain reaction (qPCR) was performed to quantify target gene copy numbers for total bacteria (16S rRNA gene) and potentially toxic *Microcystis* (*mcyE*). The 16S rRNA primer set followed López-Gutiérrez et al. (2004), and the *Microcystis*-specific *mcyE* primer set (127F/247R) followed Sipari et al. (2010). Because the extracted DNA was limited, the *mcyE* assay was applied as a targeted marker for potentially toxic *Microcystis* rather than as a comprehensive screen of all possible MC-producing cyanobacteria. For each target, recombinant plasmids containing the respective amplicon were constructed to generate standard curves. PCR products were purified using the SmartGene Gel DNA Clean-up Kit (Daejeon, South Korea), ligated into the pLUG-Prime TA-Cloning Vector Kit II (Intron Biotechnology, Seongnam, South Korea), and transformed into ECOS Competent Cells (Yeastern Biotech, New Taipei City, Taiwan). Transformants were screened by blue–white selection, cultured in LB broth, and plasmids were extracted using the Plasmid Mini Prep Kit (SmartGene, Daejeon, Korea). Plasmid DNA concentration was measured using a Qubit Flex Fluorometer (Thermo Fisher Scientific, Waltham, MA, USA), and standard curves were prepared by serial 10-fold dilutions (up to 10^7^-fold) in EB buffer.

Each 20-μl qPCR reaction contained 10 μl of 2× SYBR Green PreMix, 0.5 μl of each primer (10 μM), 2 μl of template DNA, and nuclease-free water. Negative PCR controls (no-template controls) and extraction blanks were included in all qPCR runs to monitor contamination. The thermal cycling conditions for each target gene, including initial denaturation, number of cycles, and specific annealing temperatures, are summarized in Table S3. qPCR assays were performed in triplicate for standard curves and in duplicate for samples using the CFX Duet Real-Time PCR System (Bio-Rad, Hercules, CA, USA). Standard curves showed excellent linearity (*R*^2^ > 0.992), with amplification efficiencies of 108% for 16S rRNA gene (slope = −3.1446) and 113% for *mcyE* (slope = −3.0539). Gene copy numbers in environmental samples were calculated from Cq values using the standard curve regression equations (Kim MJ et al. 2023). qPCR amplification failed for samples collected after 6 August due to insufficient template DNA, and these samples were therefore excluded from qPCR-based analyses (but retained for sequencing-based analyses where applicable).

### Statistical analysis

All statistical analyses and visualizations were performed in R (version 4.3.2; https://www.r-project.org/). Alpha diversity (Chao1, Shannon, and Simpson indices) was calculated and compared among groups using Welch’s analysis of variance (ANOVA), followed by Games–Howell post hoc comparisons. Principal coordinate analysis (PCoA) based on Bray–Curtis dissimilarity was carried out using the “phyloseq” package (ver. 1.46.0) and visualized with “ggplot2” (ver. 3.4.4). To evaluate whether the high–middle–low cyanobacteria-ratio groups reflected meaningful differences in microbial community composition, we performed permutational multivariate analysis of variance (PERMANOVA) (adonis2, 999 permutations) on Bray–Curtis dissimilarities at multiple taxonomic levels (ASV, phylum, class, and order) for both bacterial and microeukaryotic datasets. Complete PERMANOVA statistics are provided in Supplementary Table S6. For the correlation and multivariate analyses, physicochemical and microbial variables were matched using the same samples collected at each site and date. Date was included as a temporal covariate in the statistical analyses. Taxa included in the correlation analysis were first identified using Linear Discriminant Analysis Effect Size (LEfSe), and the subsequent correlations were calculated using genus-level abundance values extracted from the taxonomically aggregated bacterial and microeukaryotic community tables. Correlation analyses were conducted using Pearson’s correlation, and linear regression models were fitted to assess relationships between diversity indices and MC concentrations, with outputs visualized using “ggplot2” and “ggpubr” (ver. 0.6.0).

Pie charts, Venn diagrams, alpha diversity tables, and LEfSe analyses were produced by classifying all 56 samples into three groups (high, middle, and low; *n* = 19, 18, and 19, respectively) based on the relative abundance of Cyanobacteria (phylum). Differentially enriched taxa were identified using LEfSe implemented in the “microbiomeMarker” package (ver. 1.8.0), with parameters set to the Kruskal–Wallis test *P* < 0.01, Wilcoxon test *P* < 0.05, and linear discriminant analysis (LDA) ≥ 3.0. The LEfSe analysis was performed on taxonomically annotated community tables across hierarchical ranks from phylum to genus. For concise presentation, the main text highlights the top discriminant genera per group (Fig. 4), whereas higher-level discriminatory taxa are presented in the supplementary cladograms (Fig. S4). Because many ASVs lacked stable taxonomic annotation, ASV-level LEfSe was not used for interpretation. For genus-level comparisons, uncultured or NA taxa were excluded, and the top five discriminant genera per group were selected for visualization. Venn diagrams of ASV distributions across groups were generated using the “VennDiagram” package (ver. 1.7.3).

## Results

### Temporal dynamics of environmental factors and microbial genes

MC concentrations generally increased during the sampling period, rising from 0.266 ± 0.295 to 3.680 ± 1.850 mg/l (Fig. 1B; Table S4). qPCR analysis of total bacteria (16S rRNA gene) and potentially toxic *Microcystis* (*mcyE*) also showed increasing gene copy numbers through 6 August 2021, after which qPCR data were no longer available (Fig. 1C). During the overlapping sampling period, 16S rRNA gene abundance increased from 8.680 ± 0.661 to 9.610 ± 0.526 log copies/100 ml, and *mcyE* from 7.480 ± 1.550 to 10.100 ± 0.466 log copies/100 ml.

Water-quality and meteorological variables varied across sites and sampling dates (Table S4). DO generally exceeded 7 mg/l but temporarily declined below 5 mg/l at one site (S1) in early August, coinciding with increases in SS. pH ranged from weakly acidic to strongly alkaline (approximately 6.0–9.6), with substantial variation across sites and sampling dates. SS, BOD, and TP showed localized increases at some mid-river sites on 20 August, whereas nutrient levels remained low to moderate at most sampling points.

Spatially, upstream sites (S1–S3) showed elevated MC concentrations from late July onward, with particularly high values observed at S1 and S3 in mid- to late August (Table S4). Downstream sites (S8–S9) displayed greater temporal variability, including MC concentrations exceeding 5 mg/l at S9 on 20 August. Precipitation was limited during most sampling dates, with only minor rainfall recorded at a small number of sites, while water temperature remained relatively high (approximately 27–38°C) throughout the sampling period. These water-temperature values were measured in situ from near-surface water during daytime sampling under summer conditions and may therefore reflect strong surface heating at the time of collection. Although no single water-quality or meteorological parameter exhibited temporal trends matching those of MC concentrations or microbial gene abundances, the observed spatial and temporal variation provided the environmental context for subsequent multivariate and correlation analyses.

### Microbial community shifts associated with HABs

**Figure 2:**
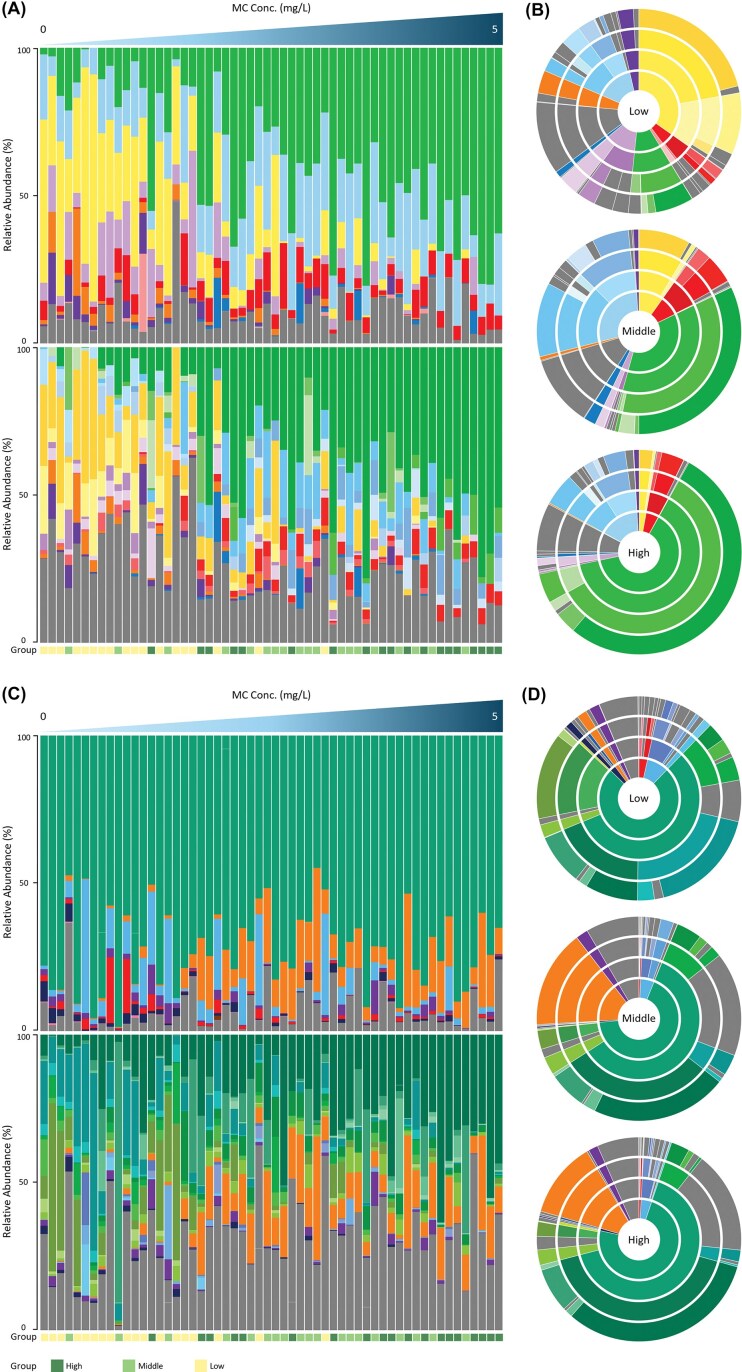
Taxonomic composition of bacterial and microeukaryotic communities across measured MC concentrations and cyanobacterial-ratio groups. (A, B) Bacterial communities and (C, D) microeukaryotic communities. (A, C) Stacked bar plots of relative abundance (%) at the phylum (top) and family (bottom) levels, ordered by increasing MC concentration. (B, D) Quadruple donut charts summarizing phylum (innermost)-to-family (outermost) composition within the low (*n* = 19), middle (*n* = 18), and high (*n* = 19) cyanobacterial-ratio groups. Color schemes for all taxa are provided in Supplementary Fig. S2.

Bacterial communities displayed observable compositional differences across the MC concentration gradient (Fig. 2A) and among cyanobacterial-ratio groups (Fig. 2B). In samples with lower MC concentrations, Proteobacteria (notably Alphaproteobacteria and Gammaproteobacteria), Actinobacteriota (Acidimicrobiia, Actinobacteria), and Bacteroidota (Bacteroidia) constituted a substantial proportion of the community, while Cyanobacteria showed comparatively low relative abundance. As MC concentrations increased, the relative abundance of Cyanobacteria increased, with compositional shifts also evident within the cyanobacterial fraction: Microcystaceae and Nostocaceae became more prominent, whereas several other cyanobacterial families showed relatively lower proportional contributions. Overall, these patterns indicate a shift from taxonomically diverse bacterial assemblages under low-MC conditions to cyanobacteria-enriched communities under high-MC conditions.

Microeukaryotic communities exhibited co-occurring but taxonomically distinct changes (Fig. 2C and 2D). In low-MC samples, diverse microeukaryotes were present, including Chlorophyta (Chlorophyceae, Trebouxiophyceae, and Volvocales), fungi (Ascomycota and Basidiomycota), and ciliates (Ciliophora). With higher MC concentrations, the relative abundance of ciliates and several Chlorophyta lineages (e.g. Chlamydomonadaceae, Volvocaceae, and Chlorellaceae) increased, while fungal taxa contributed a smaller proportion to overall community composition. Microeukaryotic communities, when examined across the same groups, exhibited distinct but co-occurring changes: fungi contributed a larger proportion in the low group, whereas ciliates and several Chlorophyta taxa (Chlorophyceae and Trebouxiophyceae) comprised higher proportions in the high group.

### Ordination and ASV distribution by MC concentration and cyanobacterial ratio

**Figure 3:**
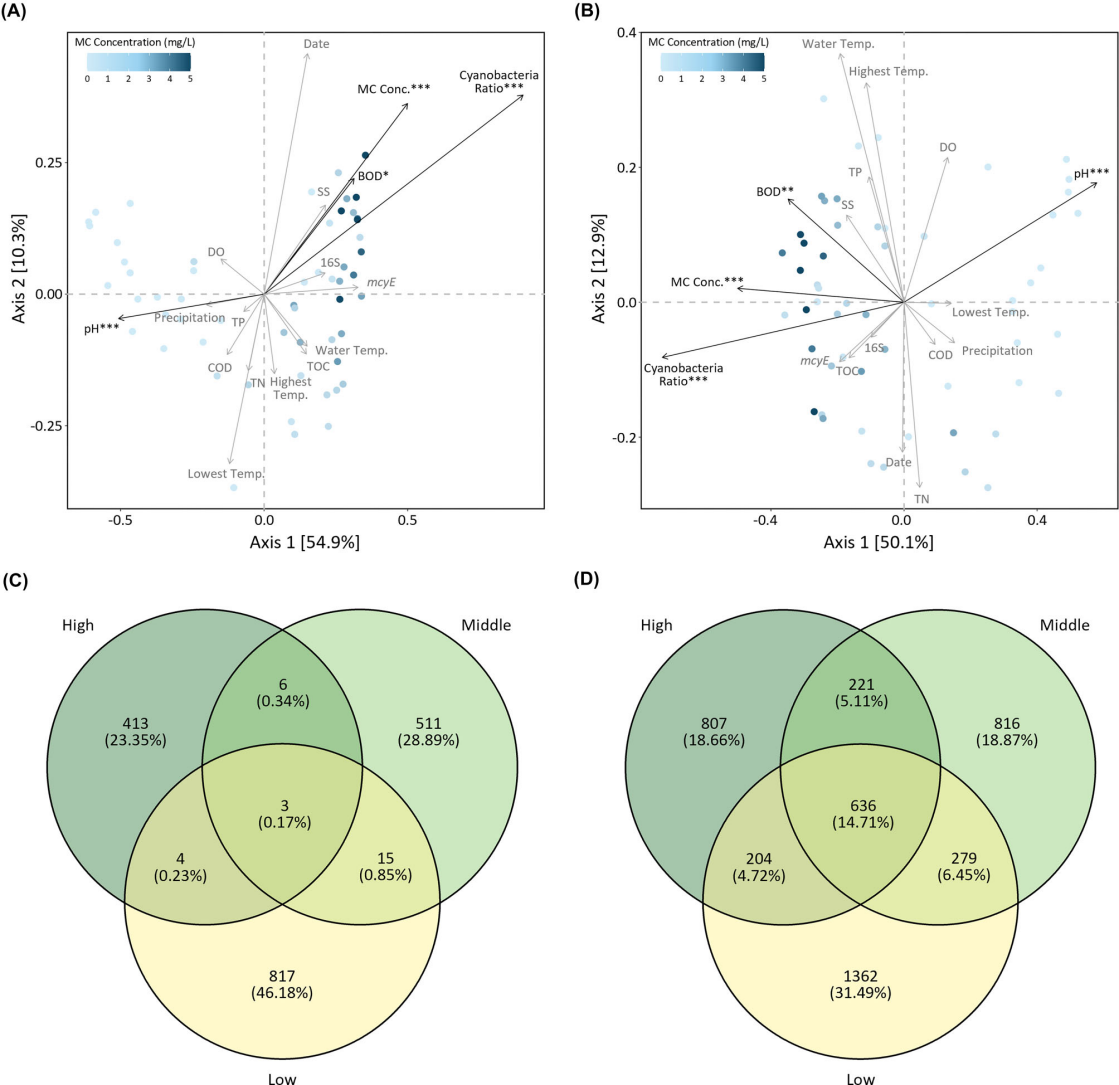
Ordination patterns and ASV distributions of bacterial and microeukaryotic communities across MC concentrations and cyanobacterial-ratio groups. (A, B) PCoA at the order level for bacterial (A) and microeukaryotic (B) communities based on Bray–Curtis dissimilarities. Points are colored according to measured MC concentration. Environmental vectors include water-quality parameters, meteorological and thermal variables, qPCR-derived gene abundances, the cyanobacterial ratio, and MC concentration. Significant vectors are shown in black, with significance levels indicated as *(*P* < 0.05), **(*P* < 0.01), and ***(*P* < 0.001). (C, D) Venn diagrams at the ASV level for bacterial (C) and microeukaryotic (D) communities showing shared and group-specific ASVs among the three cyanobacterial-ratio groups [low (*n* = 19), middle (*n* = 18), and high (*n* = 19)]. Percentages represent the proportion of total ASVs contributed by each subset.

PCoA based on Bray–Curtis dissimilarities showed observable patterns in community composition across both MC concentrations and cyanobacterial-ratio groups (Fig. 3A and 3B). Samples with higher MC concentrations tended to occupy a more compact region of the ordination space, whereas samples with lower MC concentrations were more broadly dispersed, reflecting greater compositional variability under nonbloom or transitional conditions. These trends were evident for both bacterial and microeukaryotic communities, although the degree of separation differed: bacterial communities showed a more pronounced gradient along the primary axes, whereas microeukaryotic communities exhibited a broader spread, suggesting more heterogeneous responses among microeukaryotes. Among the environmental variables evaluated, pH, MC concentration, cyanobacteria ratio, and BOD displayed significant correlations with one or more ordination axes, indicating statistical associations between these parameters and sample distribution in multivariate space.

Venn diagrams provided complementary insight into ASV distributions across the low, middle, and high groups (Fig. 3C and 3D). In bacterial communities, the low group contained the largest proportion of group-specific ASVs (46.18%), whereas only 0.17% of ASVs were shared across all three groups. Microeukaryotic communities showed a related but nonidentical structure: the low group again contained the highest proportion of unique ASVs (31.49%), whereas the high and middle groups showed similarly lower proportions of group-specific ASVs (18.66% and 18.87%, respectively). These patterns indicate distinct shifts in ASV membership across cyanobacterial-ratio groups, with a stronger reduction in shared ASVs in bacterial communities than in microeukaryotic communities. These ASV-level distinctions were consistent with PERMANOVA results (Supplementary Table S6), which showed statistically significant differences among the three groups across all tested taxonomic levels for both bacterial and microeukaryotic communities. Effect sizes varied substantially across levels, with bacterial communities showing much stronger separation at phylum-to-order levels than at the ASV level, whereas microeukaryotic communities showed more moderate but still significant differentiation across levels.

### Alpha diversity of microbial communities

Bacterial alpha diversity showed clear group-wise differences across the cyanobacterial-ratio gradient (Table 1). Under Welch’s ANOVA, Chao1, Shannon, and Simpson indices all differed significantly among groups (all *P* < 0.001), with the high group consistently showing the lowest diversity and the low group the highest. Games–Howell post hoc comparisons further indicated that all pairwise differences were significant for all three bacterial metrics, while the middle group remained intermediate between the high and low groups. When MC concentration was examined as a continuous variable, regression analyses (Fig. S3A–C) also revealed significant negative associations for all three bacterial diversity indices, indicating that higher MC levels corresponded to lower bacterial richness and evenness.

**Table 1:** Alpha diversity indices (Chao1, Shannon, and Simpson) of bacterial and microeukaryotic communities across cyanobacterial-ratio groups (high, middle, and low). Values are presented as mean ± SD, with sample sizes shown in parentheses. Differences among groups were assessed using Welch’s ANOVA, followed by Games–Howell post hoc comparisons.

	Group (mean ± SD)			Welch ANOVA F (df)	*P*	Games–Howell test		
	High (*n* = 19)	Middle (*n* = 18)	Low (*n* = 19)			High middle	High low	Middle low
Bacteria								
Chao1	23.57 ± 8.63	32.65 ± 6.86	48.20 ± 11.89	26.24 (2, 34.29)	<0.001	9.08 (*P* = 0.003)	24.63 (*P* < 0.001)	15.56 (*P* < 0.001)
Shannon	1.782 ± 0.462	2.474 ± 0.270	3.051 ± 0.253	60.09 (2, 33.94)	<0.001	0.692 (*P* < 0.001)	1.269 (*P* < 0.001)	0.577 (*P* < 0.001)
Simpson	0.662 ± 0.139	0.848 ± 0.045	0.927 ± 0.020	52.27 (2, 27.43)	<0.001	0.186 (*P* < 0.001)	0.264 (*P* < 0.001)	0.079 (*P* < 0.001)
Microeukaryota								
Chao1	226.35 ± 90.32	254.26 ± 141.82	280.16 ± 125.82	1.16 (2, 33.59)	0.327	27.91 (*P* = 0.760)	53.81 (*P* = 0.297)	25.90 (*P* = 0.828)
Shannon	3.403 ± 0.944	3.680 ± 0.665	3.961 ± 0.874	1.76 (2, 34.83)	0.187	0.277 (*P* = 0.560)	0.558 (*P* = 0.156)	0.281 (*P* = 0.518)
Simpson	0.886 ± 0.090	0.932 ± 0.031	0.921 ± 0.095	2.25 (2, 28.74)	0.123	0.047 (*P* = 0.106)	0.035 (*P* = 0.473)	−0.011 (*P* = 0.873)

In contrast, microeukaryotic alpha diversity did not show significant group-wise differences across the low, middle, and high cyanobacterial-ratio groups under Welch’s ANOVA and Games–Howell post hoc comparisons (Table 1). However, when evaluated against MC concentration as a continuous variable, regression analyses (Fig. S3D–F) revealed significant negative associations for all three diversity indices, with a weaker relationship for Chao1 and stronger relationships for Shannon and Simpson. These results suggest that, although categorical group comparisons did not detect clear contrasts among microeukaryotic communities, their alpha diversity still declined along the continuous gradient of increasing MC concentration.

### Discriminant microbial taxa across cyanobacterial-ratio groups

**Figure 4:**
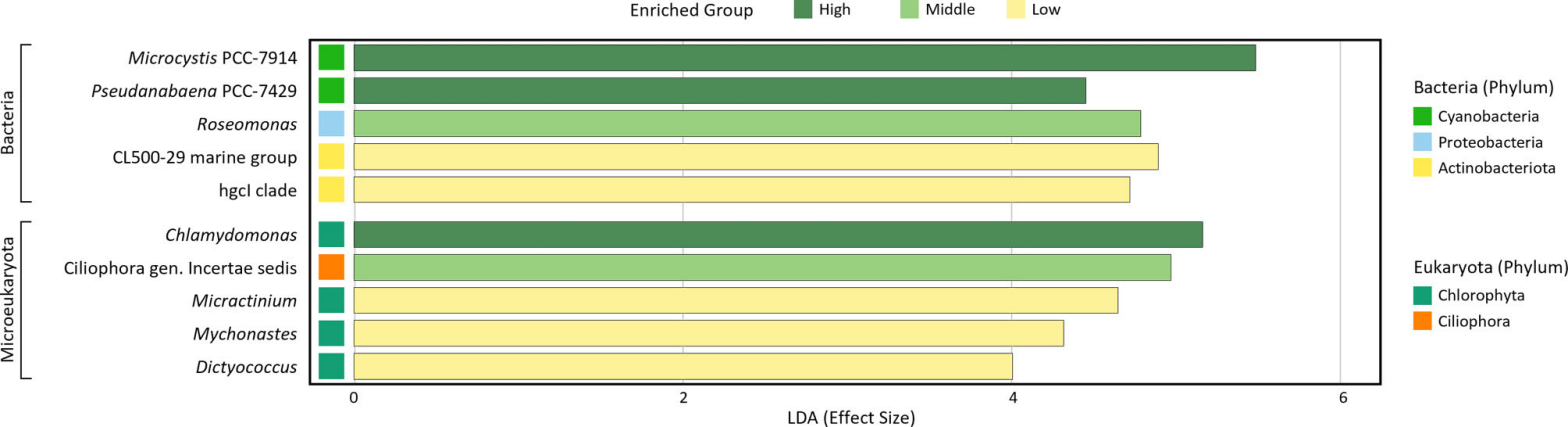
Genera differentially represented among the low (*n* = 19), middle (*n* = 18), and high (*n* = 19) cyanobacterial-ratio groups based on LEfSe analysis. Bars show LDA effect sizes for bacterial and microeukaryotic genera meeting the LEfSe significance thresholds (Kruskal–Wallis *P* < 0.01, Wilcoxon *P* < 0.05, LDA ≥ 3.0). Colors indicate the group in which each genus was enriched, and the colored blocks preceding each bar denote the corresponding phylum-level affiliation.

LEfSe analysis was used to identify taxa that were differentially represented among the low, middle, and high cyanobacterial-ratio groups (Fig. 4; Fig. S4). In the bacterial communities, two genera (CL500-29 marine group and hgcI clade) showed higher relative representation in the low group, whereas one genus (*Roseomonas*) was associated with the middle group. In the high group, cyanobacterial genera such as *Microcystis* PCC-7914 and *Pseudanabaena* PCC-7429 showed higher relative representation. At higher taxonomic resolution, low-group bacterial biomarkers were concentrated mainly in Actinobacteriota- and Firmicutes-related lineages, whereas middle-group biomarkers were more frequently assigned to Proteobacteria- and Bacteroidota-associated lineages; the high group was primarily represented by the two cyanobacterial lineages noted above (Fig. S4A).

Microeukaryotic communities also exhibited group-specific associations. Three Chlorophyta genera (*Micractinium, Mychonastes*, and *Dictyococcus*) showed greater representation in the low group, whereas the high group was characterized by higher relative representation of *Chlamydomonas*. In addition, one ciliate lineage (Ciliophora gen. incertae sedis) was identified as a middle-group indicator under the LEfSe thresholds applied (Kruskal–Wallis *P* < 0.01; LDA ≥ 3.0). These LEfSe-based patterns, which were also evident from kingdom to genus levels in the cladograms (Fig. S4), identified the taxa most strongly associated with each cyanobacterial-ratio group and provided the basis for the subsequent correlation analysis.

### Correlation patterns among environmental and microbial variables

**Figure 5:**
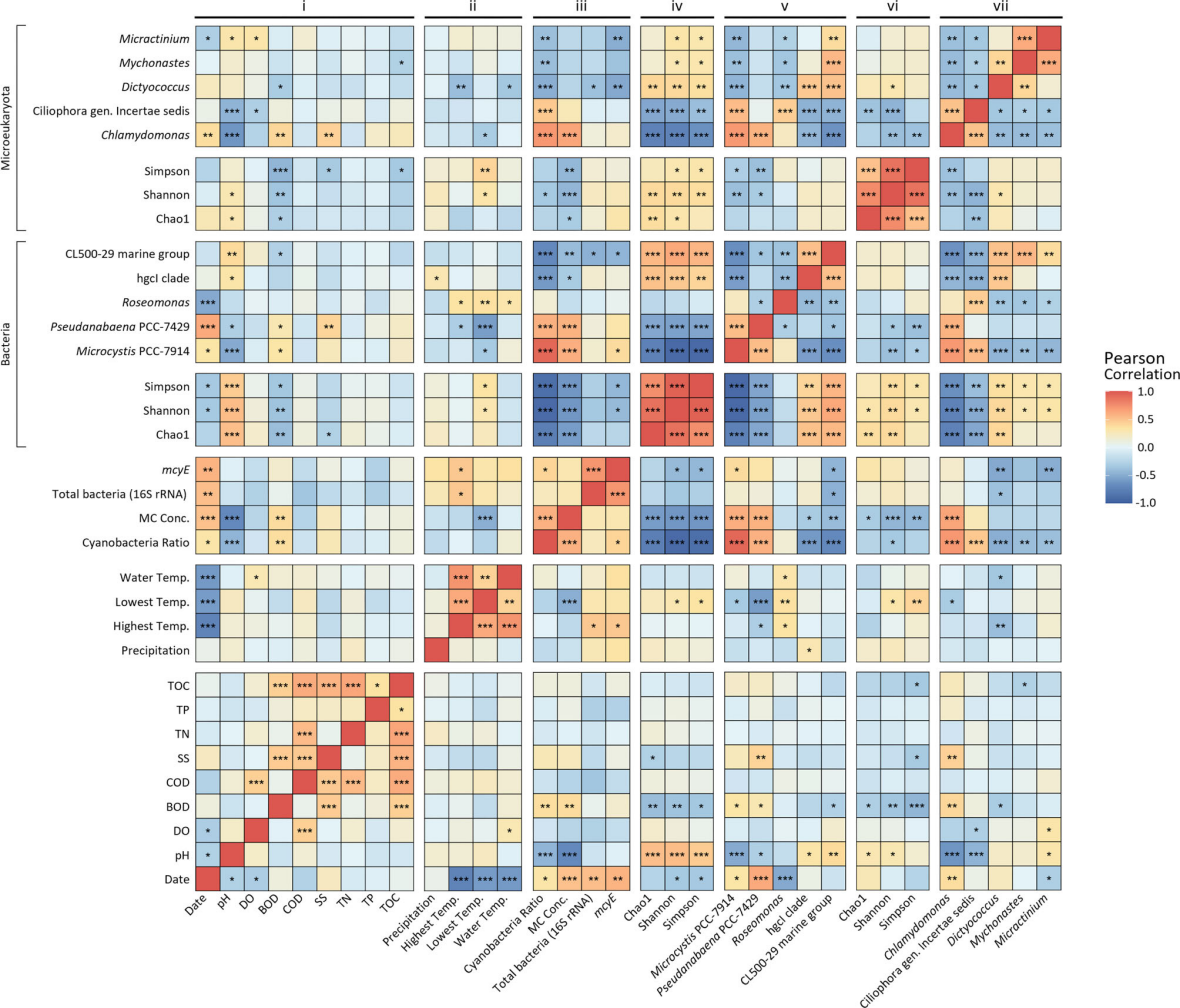
Pearson correlation heatmap integrating water-quality variables, bloom-related indicators, and microbial community metrics. The heatmap displays Pearson correlation coefficients among seven parameter categories: (i) water-quality variables (pH, DO, BOD, COD, SS, TN, TP, and TOC); (ii) meteorological and thermal conditions (precipitation, highest and lowest air temperature, and water temperature); (iii) bloom-related indicators (MC concentration, cyanobacterial ratio, qPCR-based 16S rRNA gene and *mcyE*); (iv) bacterial alpha-diversity indices (Chao1, Shannon, and Simpson); (v) bacterial genera identified as differentially represented among cyanobacterial-ratio groups by LEfSe; (vi) microeukaryotic alpha-diversity indices; and (vii) microeukaryotic genera enriched by LEfSe analysis. Significance levels for correlation coefficients are indicated as *(*P* < 0.05), **(*P* < 0.01), and ***(*P* < 0.001), with nonsignificant correlations shown without asterisks.

Correlation analysis integrating physicochemical parameters, HAB-related indicators, and microbial metrics revealed several coherent and biologically meaningful patterns (Fig. 5). Shannon diversity emerged as the most responsive alpha-diversity index in both domains, showing negative correlations with both MC concentration (r = −0.608, *P* < 0.001) and cyanobacterial ratio (r = −0.908, *P* < 0.001) in bacterial communities, and weaker but still significant negative correlations with MC concentration (r = −0.432, *P* < 0.001) and cyanobacterial ratio (r = −0.322, *P* = 0.015) in microeukaryotic communities. These results suggest that bloom intensity is associated with a stronger diversity response in bacterial communities, whereas microeukaryotic diversity responds more gradually and heterogeneously.

Beyond reductions in diversity, bloom-associated taxa induced pronounced shifts in the taxonomic structure of both communities. Using genus-level abundance values, *Microcystis* and *Pseudanabaena*—two cyanobacterial genera identified by LEfSe as enriched in the high group—were both positively correlated with *Chlamydomonas* (r = 0.701 and 0.574, respectively; both *P* < 0.001). In addition, *Microcystis* was positively correlated with the middle-group ciliate lineage (Ciliophora gen. incertae sedis; r = 0.533, *P* < 0.001) and negatively correlated with taxa characteristic of the low group, including CL500-29 marine group, hgcI clade, *Micractinium, Mychonastes*, and *Dictyococcus* (r = −0.653 to −0.373, all *P* < 0.01). These contrasting correlations suggest a clear separation between taxa characteristic of low versus high conditions, with bloom-associated bacterial and microeukaryotic taxa showing positive associations with each other and inverse associations with several low-group taxa.

Environmental parameters also exhibited distinct associations with bloom indicators and community responses. pH showed strong negative correlations with MC concentration (r = −0.695, *P* < 0.001), cyanobacterial ratio (r = −0.439, *P* < 0.001), and *Microcystis* abundance (r = −0.497, *P* < 0.001). In contrast, BOD showed modest positive correlations with MC concentration (r = 0.363, *P* = 0.006) and Microcystis abundance (r = 0.317, *P* = 0.017), indicating increased oxygen demand during bloom periods rather than a generalized increase in organic matter. Other water-quality variables, including SS, TN, TP, and TOC, showed weaker or nonsignificant relationships with MC concentration in this dataset. Collectively, these environmental–biological correlations indicate that bloom intensification in this system was most strongly associated with pH decline, increased oxygen demand, and coordinated shifts in bacterial and microeukaryotic taxa.

## Discussion

HABs in freshwater systems arise from interactions among hydrological conditions, physicochemical variability, and microbial community dynamics (Kim MJ et al. 2023, Kim et al. 2025). Although previous studies have largely characterized bloom events through cyanobacterial abundance or microcystin (MC) concentrations (Kim et al. 2020, Villalobos et al. 2025), fewer have evaluated how bacterial and microeukaryotic communities jointly reorganize as blooms intensify, particularly in regulated river systems. This gap is relevant in the Nakdong River, where weir construction has increased hydraulic residence time, promoted organic matter accumulation, and enhanced bloom susceptibility in ways distinct from lentic environments (Kim J et al. 2023; Ministry of Environment 2016). By integrating environmental parameters, toxin levels, and cross-domain microbial profiling during a bloom-affected period in the Nakdong River, this study provides a system-level view of bloom-associated ecological restructuring in a regulated riverine ecosystem. Although direct standing-stock indicators such as chlorophyll-a concentration or cyanobacterial cell density were not measured from the same samples, nearby public algal monitoring data (Supplementary Table S5) showed elevated harmful cyanobacterial and *Microcystis* cell densities at representative mid- and downstream stations during the study period, supporting the interpretation that the sampled sites were observed under bloom-associated conditions.

Both bacterial and microeukaryotic communities exhibited distinct compositional transitions aligned with increasing MC concentrations and higher cyanobacterial ratios (Fig. 2). Under low-MC conditions, bacterial communities were dominated by heterotrophic Actinobacteriota and Proteobacteria—taxa involved in organic matter processing under relatively stable conditions (Xu et al. 2018, Jeon et al. 2024). As bloom intensity increased, Cyanobacteria, particularly Microcystaceae and related cyanobacterial lineages, became predominant. Microeukaryotic communities also shifted from diverse assemblages of Chlorophyta, fungi, and ciliates toward increased representation of Chlorophyta (e.g. Chlamydomonadaceae and Volvocaceae) and Ciliophora. These coordinated shifts indicate that bloom intensification acted as a strong ecological filter across both bacterial and microeukaryotic communities, reducing diversity while favoring bloom-associated taxa. The steeper decline in bacterial alpha diversity, compared with the more moderate response in microeukaryotic communities, suggests that bacterial communities were more strongly compressed along the bloom gradient, whereas microeukaryotic responses remained relatively heterogeneous. Shannon diversity emerged as the most responsive alpha-diversity metric across domains, reflecting synchronous losses in richness and evenness and functioning as a potential sentinel indicator of bloom-associated ecological compression. These patterns further support the interpretation that bloom intensification compressed microbial diversity while selectively favoring bloom-associated taxa.

Discriminant taxa identified through LEfSe analysis further revealed asymmetric restructuring across the cyanobacterial gradient (Fig. 4; Fig. S4). The low group was enriched in the CL500-29 marine group and hgcI clade (Actinobacteriota), whereas the high group was enriched in bloom-associated cyanobacterial taxa, including *Microcystis* PCC-7914 and *Pseudanabaena* PCC-7429. The middle group showed a more limited but still detectable signature, including one enriched bacterial genus (*Roseomonas*) and one enriched microeukaryotic lineage (Ciliophora gen. incertae sedis) under the applied thresholds. Rather than indicating a strictly binary structure, these patterns suggest that the middle group represented a more limited and potentially transitional ecological state compared with the more strongly differentiated low and high groups. Microeukaryotic communities showed complementary group-specific associations, with *Micractinium, Mychonastes*, and *Dictyococcus* enriched in the low group, *Chlamydomonas* enriched in the high group, and a ciliate lineage (Ciliophora gen. incertae sedis) associated with the middle group. These LEfSe-based patterns, which were also evident from kingdom to genus levels in the cladograms (Fig. S4), support the view that HAB intensification reorganizes cross-domain communities asymmetrically rather than uniformly along the cyanobacterial gradient (Mankiewicz-Boczek and Font-Nájera 2022, Crevecoeur et al. 2023).

Correlation analyses further clarified how environmental conditions and bloom indicators interact with microbial diversity and taxonomic structure (Fig. 5). In this dataset, pH showed strong negative correlations with MC concentration, cyanobacterial ratio, and *Microcystis* abundance, whereas BOD showed modest positive associations with bloom indicators. This apparent mismatch between high MC concentrations and neutral to weakly acidic pH does not necessarily contradict bloom-associated conditions. Elevated pH is typically linked to periods of intense cyanobacterial photosynthesis, whereas dissolved MC can remain high after bloom senescence or cell lysis, when microbial decomposition and respiration may lower pH and increase oxygen demand (McKindles et al. 2020, Zepernick et al. 2021). In a regulated river such as the Nakdong, short-term hydrological disturbances, including rainfall-driven transitions between sampling dates, may further accelerate post-bloom-like physicochemical and microbial shifts; thus, some late-period samples may reflect transitional or decay-phase bloom conditions rather than a contemporaneous peak-growth state (Kang et al. 2024). The observed correlation structure supports the presence of a bloom-associated assemblage centered on key cyanobacterial and microeukaryotic taxa, including *Microcystis, Pseudanabaena*, and *Chlamydomonas*, while the middle-group ciliate lineage (Ciliophora gen. incertae sedis) occupied an intermediate but positively associated position within this correlation structure. Notably, this pattern did not reflect a uniform decline of all chlorophytes. Instead, the opposite responses of *Chlamydomonas* versus low-group chlorophytes such as *Micractinium* and *Mychonastes* suggest lineage-specific ecological strategies within Chlorophyta under bloom-associated conditions (Deblois et al. 2013, Soares et al. 2013). Low-group taxa (e.g. CL500-29 marine group, hgcI clade, *Micractinium*, and *Mychonastes*) were inversely associated with bloom indicators and bloom-associated taxa, suggesting coordinated ecological reorganization during bloom development rather than simple community turnover. These structured correlations, together with the markedly reduced shared ASV fraction in bacteria (0.17%) and the more moderate shared fraction in microeukaryota (14.71%) across groups (Fig. 3), indicate that bloom development reorganized microbial communities at both intradomain and interdomain levels.

These findings have several ecological implications. Although reduced diversity and dominance of bloom-forming taxa are expected outcomes under HAB conditions, our results suggest that bloom-associated restructuring within microeukaryotes was not uniform. In particular, the positive association of Chlamydomonas contrasted with the inverse associations observed for other chlorophytes such as Micractinium and Mychonastes, indicating lineage-specific responses within Chlorophyta (Deblois et al. 2013, Soares et al. 2013). This pattern suggests that cyanobacterial blooms may favor selected green algal lineages rather than uniformly suppressing all chlorophytes, although further work is needed to determine whether this represents a generalizable ecological pattern or a context-dependent feature of the present bloom episode (Deblois et al. 2013, Soares et al. 2013). Declines in bacterial diversity may alter functional redundancy and nutrient cycling during bloom development (Louca et al. 2018). Although bloom-associated ciliate lineages increased under bloom conditions, shifts in community composition may still constrain grazing efficiency, particularly if dominant ciliates are less effective against colony-forming or mucilaginous Cyanobacteria. In the lower Nakdong River, where multiple weirs and prolonged residence time create impounded, slow-flowing conditions favorable for cyanobacterial proliferation (Ha et al. 1999, Bae et al. 2018, Park et al. 2021, Lee et al. 2024), the co-occurrence of Microcystis and Chlamydomonadaceae, including Chlamydomonas, observed here is consistent with bloom-associated restructuring under hydrologically stagnant conditions. Similar positive covariance between Microcystis and Chlamydomonas has also been reported in another bloom-affected aquatic system (Lehman et al. 2021). The co-occurrence of selected chlorophyte lineages and Cyanobacteria may therefore complicate predictions of bloom trajectories and toxin dynamics.

Despite the insights gained, several limitations warrant consideration. Our sampling window covered a single three-week bloom period, limiting assessment of seasonal or interannual variability. Amplicon sequencing provided taxonomic resolution but limited functional inference, which could be addressed by future metagenomic or metatranscriptomic analyses. Nonetheless, the consistency of cross-domain restructuring across sites supports the robustness of the observed patterns. Taxa such as *Microcystis* and *Pseudanabaena*, which were directly associated with cyanobacterial bloom-related conditions in this study, may serve as practical bioindicators to complement physicochemical monitoring. By contrast, Chlamydomonas and bloom-associated ciliate lineages should be interpreted more cautiously as taxa associated with bloom-related community restructuring at the sampled sites, rather than as general indicators of cHABs. For regulated rivers such as the Nakdong, management should prioritize nutrient-load reduction and restoration of flow regimes, while incorporating microbial indicators identified herein to strengthen HAB risk assessment and mitigation strategies.

This study shows that bloom-associated conditions in a regulated river system were accompanied by rapid and parallel restructuring of bacterial and microeukaryotic communities. By integrating toxin measurements, hydrological and biogeochemical variables, and amplicon-based profiling, we observed that bloom intensification was associated with reduced microbial diversity, collapse of low-ratio bacterial and chlorophyte assemblages, and coordinated increases in *Microcystis, Pseudanabaena*, and selected green algal and ciliate lineages. These cross-domain patterns were observed in the lower Nakdong River, a regulated river setting with impounded, slow-flowing hydrological features. Our findings provide ecological insight into bloom-associated microbial restructuring at the sampled river locations and highlight the value of integrating microbial and physicochemical indicators when assessing ecosystem change under increasing HAB pressure.

## Supplementary Material

fiag057_Supplemental_File

## Data Availability

Raw amplicon reads (16S rRNA gene V4 and ITS2 region) are deposited in the NCBI Sequence Read Archive under BioProject PRJNA1334184 (https://www.ncbi.nlm.nih.gov/bioproject/PRJNA1334184). Samples are registered in NCBI BioSample as SAMN51837082–SAMN51837138 (16S rRNA gene) and SAMN51837142–SAMN51837198 (ITS2 region), and the BioSample Sample Name fields (e.g. S1-1_16S, S1-1_ITS2) match the identifiers used in the manuscript. Metadata follow the MIxS/MIMARKS-survey (water) v6.0 standard.
