# Supplementary material for: Bacterial and microeukaryotic assemblage transitions during a harmful algal bloom in the Nakdong River
Source: FEMS Microbiol Ecol. 2026 Jun 2;102(7):fiag057. doi: 10.1093/femsec/fiag057 (PMC13278487; doi:10.1093/femsec/fiag057)
Supplement: fiag057_Supplemental_File [file fiag057_supplemental_file.docx]

**Table S1.** Spatial-temporal information of freshwater samples.

| Site label | Location | | Collection date |
| --- | --- | --- | --- |
|  | Name | GPS |  |
| S1 | Gangjeong-Goryeong Weir | 35.844281, 128.444749 | 28/07/2021 |
|  |  |  | 30/07/2021 |
|  |  |  | 04/08/2021 |
|  |  |  | 06/08/2021 |
|  |  |  | 11/08/2021 |
|  |  |  | 20/08/2021 |
| S2 | Hwawon Amusement Park | 35.810999, 128.476563 | 28/07/2021 |
|  |  |  | 30/07/2021 |
|  |  |  | 04/08/2021 |
|  |  |  | 06/08/2021 |
|  |  |  | 11/08/2021 |
|  |  |  | 13/08/2021 |
| S3 | Goryeong Bridge | 35.752617, 128.386493 | 28/07/2021 |
|  |  |  | 30/07/2021 |
|  |  |  | 04/08/2021 |
|  |  |  | 06/08/2021. |
|  |  |  | 11/08/2021 |
|  |  |  | 13/08/2021 |
|  |  |  | 20/08/2021 |
| S4 | Dodongseowon | 35.702791, 128.371262 | 28/07/2021 |
|  |  |  | 30/07/2021 |
|  |  |  | 04/08/2021 |
|  |  |  | 06/08/2021 |
|  |  |  | 11/08/2021 |
|  |  |  | 13/08/2021 |
|  |  |  | 20/08/2021 |
| S5 | National Industrial Complex Intake Station | 35.634806, 128.403357 | 28/07/2021 |
|  |  |  | 30/07/2021 |
|  |  |  | 04/08/2021 |
|  |  |  | 06/08/2021 |
|  |  |  | 11/08/2021 |
|  |  |  | 13/08/2021 |
|  |  |  | 20/08/2021 |
| S6 | Ibang Pumping Station | 35.6104596, 128.3603572 | 28/07/2021 |
|  |  |  | 30/07/2021 |
|  |  |  | 04/08/2021 |
|  |  |  | 06/08/2021 |
| S7 | Hapcheon Weir | 35.593484, 128.357026 | 28/07/2021 |
|  |  |  | 30/07/2021 |
|  |  |  | 04/08/2021 |
|  |  |  | 06/08/2021 |
|  |  |  | 11/08/2021 |
|  |  |  | 13/08/2021 |
| S8 | Haman Weir | 35.378165, 128.551053 | 28/07/2021 |
|  |  |  | 30/07/2021 |
|  |  |  | 04/08/2021 |
|  |  |  | 06/08/2021 |
|  |  |  | 11/08/2021 |
|  |  |  | 13/08/2021 |
|  |  |  | 20/08/2021 |
| S9 | Bonpo Intake Station | 35.3714576, 128.6488982 | 28/07/2021 |
|  |  |  | 30/07/2021 |
|  |  |  | 04/08/2021 |
|  |  |  | 06/08/2021 |
|  |  |  | 11/08/2021 |
|  |  |  | 13/08/2021 |
|  |  |  | 20/08/2021 |

**Table S2.** Sequencing information.

| Target gene | Primer | Sequences (5’-3’) | Tm temp. (℃) |
| --- | --- | --- | --- |
| 16S rRNA V4 region | XT-V4-F | ACACTCTTTCCCTACACGACGCTCTTC | 73.4 |
|  | XT-V4-R | GTGACTGGAGTTCAGACGTGTGCTCTT | 67.8 |
| ITS region | ITS1-F | TCGGTAGGTGAACCTGCGG | 59.5 |
|  | ITS4-R | TCCTCCGCTTATTGATATGC | 52.1 |

**Table S3.** Primers used for qPCR and thermal cycling conditions

| Target gene | Primer / Probe | Sequences (5' - 3') | Condition |
| --- | --- | --- | --- |
| Total Bacteria (16S rRNA) | 16S_F | CCTACGGGAGGCAGCAG | 94°C 5 m, 35ｘ(94°C 30 s,  55°C 45 s, 72°C 45 s), 72°C 10 m |
|  | 16S_R | AATCCGCGGCTGGCA |  |
| MC-producing Microcystis (*mcyE*) | 127F | AAGCAAACTGCTCCCGGTATC | 95°C 15 m, 40ｘ(95°C 15 s,  62°C 60 s) |
|  | 247R | CAATGGGAGCATAACGAGTCAA |  |

**Table S4.** Physicochemical water quality parameters, meteorological and thermal conditions, and microcystin (MC) concentrations measured at sampling sites (S1–S9) during the study period. Abbreviations: DO, dissolved oxygen (mg/L); BOD, biochemical oxygen demand (mg/L); COD, chemical oxygen demand (mg/L); SS, suspended solids (mg/L); TN, total nitrogen (mg/L); TP, total phosphorus (mg/L); TOC, total organic carbon (mg/L); Precipitation (mm); Highest/Lowest Temp., daily maximum/minimum air temperature (°C); Water Temp., surface water temperature (°C); MC Conc., microcystin concentration (μg/L).

| Site | Date | Water Quality | | | | | | | | Meteorological & Thermal Conditions | | | | *MC Conc. (μg/L) |  |
| --- | --- | --- | --- | --- | --- | --- | --- | --- | --- | --- | --- | --- | --- | --- | --- |
|  |  | pH | DO (mg/L) | BOD (mg/L) | COD (mg/L) | SS (mg/L) | TN (mg/L) | TP (mg/L) | TOC (mg/L) | Precipitation (mm) | Lowest Temp. (°C) | Highest Temp. (°C) | Water Temp. (°C) |  |  |
|  |  |  |  |  |  |  |  |  |  |  |  |  |  |  |  |
| S1 | 28/07/2021 | 7.55 | 6.5 | 1.5 | 6.1 | 2.8 | 1.932 | 0.042 | 3.9 | 0.0 | 22.9 | 33.6 | 30.5 | 139.3 |  |
|  | 30/07/2021 | 7.45 | 6.8 | 1.4 | 6.3 | 2.8 | 1.939 | 0.041 | 4.0 | 0.0 | 24.6 | 35.3 | 31.5 | 157.9 |  |
|  | 04/08/2021 | 7.51 | 4.4 | 2.1 | 6.2 | 7.2 | 1.682 | 0.043 | 3.8 | 0.0 | 24.6 | 34.9 | 31.0 | 238.0 |  |
|  | 06/08/2021 | 9.24 | 4.3 | 2.0 | 6.1 | 7.1 | 1.671 | 0.047 | 3.8 | 0.0 | 25.2 | 36.4 | 32.3 | 0.27 |  |
|  | 11/08/2021 | 8.93 | 6.8 | 2.2 | 6.4 | 4.8 | 1.526 | 0.048 | 3.5 | 2.0 | 21.2 | 30.7 | 28.7 | 170.2 |  |
|  | 20/08/2021 | 5.98 | 6.8 | 1.8 | 6.0 | 5.2 | 1.723 | 0.084 | 3.8 | 0.0 | 20.3 | 30.3 | 28.5 | >5,000 |  |
| S2 | 28/07/2021 | 7.92 | 8.3 | 3.0 | 6.8 | 5.2 | 1.751 | 0.084 | 3.2 | 0.0 | 22.9 | 33.6 | 30.5 | 228.9 |  |
|  | 30/07/2021 | 8.21 | 9.5 | 3.2 | 7.6 | 7.6 | 3.020 | 0.064 | 2.7 | 0.0 | 24.6 | 35.3 | 31.5 | 20.6 |  |
|  | 04/08/2021 | 8.58 | 9.5 | 3.1 | 7.8 | 6.2 | 1.841 | 0.064 | 3.8 | 0.0 | 24.6 | 34.9 | 31.0 | 5.6 |  |
|  | 06/08/2021 | 7.71 | 6.8 | 2.4 | 6.8 | 7.6 | 2.514 | 0.064 | 3.5 | 0.0 | 25.2 | 36.4 | 32.3 | 715.0 |  |
|  | 11/08/2021 | 7.90 | 8.4 | 1.8 | 6.2 | 4.8 | 1.875 | 0.075 | 3.7 | 2.0 | 21.2 | 30.7 | 28.7 | 40.8 |  |
|  | 13/08/2021 | 8.24 | 9.5 | 1.2 | 7.5 | 3.6 | 1.642 | 0.072 | 3.8 | 0.0 | 23.0 | 29.0 | 29.2 | ND |  |
| S3 | 28/07/2021 | 7.86 | 7.0 | 1.5 | 6.9 | 10.5 | 2.181 | 0.052 | 3.2 | 0.0 | 22.4 | 35.0 | 30.7 | 111.4 |  |
|  | 30/07/2021 | 8.15 | 9.4 | 3.0 | 9.0 | 11.8 | 2.625 | 0.068 | 4.1 | 0.0 | 24.5 | 34.3 | 32.8 | 65.6 |  |
|  | 04/08/2021 | 9.04 | 7.1 | 2.5 | 7.5 | 7.4 | 2.039 | 0.059 | 3.6 | 0.0 | 24.0 | 34.3 | 30.3 | 29.4 |  |
|  | 06/08/2021 | 8.77 | 8.2 | 2.9 | 7.6 | 7.4 | 3.025 | 0.063 | 3.7 | 5.0 | 24.1 | 36.2 | 31.3 | 5.00 |  |
|  | 11/08/2021 | 8.92 | 7.1 | 1.6 | 7.1 | 6.4 | 2.184 | 0.053 | 3.4 | 0.0 | 21.4 | 30.9 | 29.1 | 246.5 |  |
|  | 13/08/2021 | 7.63 | 7.1 | 1.6 | 7.1 | 6.4 | 1.854 | 0.054 | 3.0 | 0.0 | 23.5 | 29.7 | 29.2 | 154.1 |  |
|  | 20/08/2021 | 7.10 | 7.8 | 1.3 | 6.7 | 11.8 | 2.224 | 0.073 | 3.4 | 0.0 | 19.7 | 28.9 | 28.8 | 3462.9 |  |
| S4 | 28/07/2021 | 7.68 | 8.8 | 2.4 | 9.0 | 6.5 | 4.254 | 0.131 | 7.8 | 0.0 | 22.4 | 35.0 | 31.5 | 28.5 |  |
|  | 30/07/2021 | 8.94 | 8.2 | 1.7 | 9.3 | 6.4 | 4.207 | 0.127 | 7.5 | 0.0 | 24.5 | 34.3 | 30.8 | 23.8 |  |
|  | 04/08/2021 | 7.72 | 8.3 | 3.1 | 8.7 | 7.6 | 4.855 | 0.168 | 7.9 | 0.0 | 24.0 | 34.3 | 31.2 | 88.9 |  |
|  | 06/08/2021 | 8.62 | 8.2 | 1.7 | 9.3 | 6.4 | 4.207 | 0.127 | 7.5 | 5.0 | 24.1 | 36.2 | 32.3 | 3.48 |  |
|  | 11/08/2021 | 8.27 | 8.4 | 1.2 | 7.8 | 5.2 | 4.615 | 0.086 | 5.8 | 0.0 | 21.4 | 30.9 | 29.5 | 118.0 |  |
|  | 13/08/2021 | 8.40 | 8.4 | 1.2 | 7.8 | 5.2 | 4.615 | 0.086 | 5.8 | 0.0 | 23.5 | 29.7 | 29.1 | 982.4 |  |
|  | 20/08/2021 | 6.92 | 7.9 | 6.2 | 12.3 | 134.0 | 2.992 | 0.219 | 11.0 | 0.0 | 19.7 | 28.9 | 29.3 | 3372.7 |  |
| S5 | 28/07/2021 | 8.32 | 8.5 | 2.3 | 6.7 | 7.2 | 2.630 | 0.075 | 5.0 | 0.0 | 21.9 | 34.3 | 32.5 | 428.9 |  |
|  | 30/07/2021 | 8.42 | 9.2 | 3.4 | 8.7 | 8.4 | 2.840 | 0.060 | 6.0 | 0.0 | 22.9 | 34.4 | 32.2 | 201.2 |  |
|  | 04/08/2021 | 8.67 | 9.2 | 3.4 | 8.7 | 8.4 | 2.840 | 0.050 | 5.3 | 0.0 | 23.0 | 34.5 | 31.6 | 30.4 |  |
|  | 06/08/2021 | 7.08 | 8.1 | 1.7 | 5.8 | 6.4 | 2.510 | 0.086 | 4.0 | 0.0 | 22.7 | 37.1 | 33.3 | 4914.4 |  |
|  | 11/08/2021 | 6.82 | 7.6 | 3.5 | 7.9 | 7.5 | 3.510 | 0.084 | 4.5 | 0.0 | 21.2 | 31.1 | 30.9 | >5,000 |  |
|  | 13/08/2021 | 7.20 | 8.8 | 1.3 | 5.6 | 5.7 | 2.054 | 0.091 | 4.8 | 0.0 | 22.5 | 28.7 | 29.1 | 886.9 |  |
|  | 20/08/2021 | 6.80 | 8.0 | 2.7 | 6.3 | 31.2 | 2.344 | 0.099 | 4.5 | 0.0 | 20.1 | 29.5 | 30.2 | >5,000 |  |
| S6 | 28/07/2021 | 9.21 | 10.5 | 1.3 | 8.5 | 12.0 | 2.154 | 0.049 | 4.1 | 0.0 | 21.9 | 34.3 | 32.5 | 0.21 |  |
|  | 30/07/2021 | 9.35 | 7.9 | 1.9 | 8.2 | 13.0 | 2.451 | 0.068 | 3.9 | 0.0 | 22.9 | 34.4 | 32.2 | 0.60 |  |
|  | 04/08/2021 | 8.84 | 7.5 | 1.2 | 6.9 | 7.4 | 2.196 | 0.072 | 3.5 | 0.0 | 23.0 | 34.5 | 31.6 | 1.00 |  |
|  | 06/08/2021 | 8.84 | 7.6 | 1.8 | 7.3 | 10.0 | 2.257 | 0.057 | 3.7 | 0.0 | 22.7 | 37.1 | 33.3 | 0.84 |  |
| S7 | 28/07/2021 | 8.31 | 7.3 | 2.0 | 7.2 | 5.3 | 2.024 | 0.530 | 4.2 | 0.0 | 21.9 | 34.3 | 32.5 | 1.19 |  |
|  | 30/07/2021 | 9.78 | 9.8 | 3.5 | 8.1 | 10.0 | 2.178 | 0.052 | 5.0 | 0.0 | 22.9 | 34.4 | 32.2 | 1.26 |  |
|  | 04/08/2021 | 8.49 | 9.7 | 2.8 | 7.8 | 6.4 | 2.055 | 0.044 | 4.5 | 0.0 | 23.0 | 34.5 | 31.6 | 1.07 |  |
|  | 06/08/2021 | 8.21 | 9.6 | 2.7 | 7.3 | 6.6 | 1.957 | 0.048 | 4.2 | 0.0 | 22.7 | 37.1 | 33.3 | 555.7 |  |
|  | 11/08/2021 | 8.33 | 6.8 | 1.5 | 6.4 | 4.8 | 1.882 | 0.062 | 3.9 | 0.0 | 21.2 | 31.1 | 30.9 | 4.90 |  |
|  | 13/08/2021 | 7.98 | 6.9 | 1.2 | 6.5 | 4.3 | 1.752 | 0.052 | 4.1 | 0.0 | 22.5 | 28.7 | 29.1 | 1.23 |  |
| S8 | 28/07/2021 | 8.56 | 8.7 | 2.5 | 6.8 | 25.0 | 1.984 | 0.062 | 4.2 | 0.0 | 22.9 | 33.3 | 31.7 | 682.0 |  |
|  | 30/07/2021 | 9.57 | 8.5 | 2.8 | 7.4 | 48.0 | 2.197 | 0.064 | 5.2 | 0.0 | 23.0 | 34.4 | 36.3 | 8.2 |  |
|  | 04/08/2021 | 7.14 | 7.2 | 3.2 | 7.8 | 54.0 | 2.514 | 0.084 | 5.6 | 0.0 | 24.3 | 34.5 | 33.9 | 1555.3 |  |
|  | 06/08/2021 | 9.63 | 8.5 | 2.8 | 7.4 | 48.0 | 2.197 | 0.064 | 5.2 | 0.0 | 22.9 | 35.9 | 37.9 | 80.6 |  |
|  | 11/08/2021 | 9.42 | 8.8 | 2.0 | 6.3 | 6.4 | 1.824 | 0.055 | 3.9 | 0.0 | 21.5 | 31.1 | 28.9 | 0.24 |  |
|  | 13/08/2021 | 8.98 | 7.4 | 3.0 | 7.5 | 50.0 | 2.358 | 0.077 | 5.3 | 0.5 | 22.8 | 28.5 | 26.8 | 657.4 |  |
|  | 20/08/2021 | 8.94 | 7.6 | 1.4 | 6.1 | 12.0 | 1.936 | 0.076 | 3.6 | 0.0 | 19.6 | 30.4 | 27.8 | 255.8 |  |
| S9 | 28/07/2021 | 7.51 | 9.5 | 2.6 | 6.4 | 8.5 | 1.765 | 0.071 | 4.3 | 0.0 | 22.2 | 33.8 | 36.2 | 774.5 |  |
|  | 30/07/2021 | 7.51 | 10.7 | 2.5 | 7.7 | 10.8 | 1.902 | 0.043 | 5.0 | 0.0 | 22.9 | 34.0 | 36.2 | 362.5 |  |
|  | 04/08/2021 | 7.54 | 10.3 | 2.8 | 7.3 | 11.2 | 1.454 | 0.034 | 4.8 | 0.0 | 24.1 | 35.4 | 34.3 | 1201.0 |  |
|  | 06/08/2021 | 6.69 | 6.5 | 1.9 | 6.6 | 15.2 | 1.645 | 0.083 | 4.0 | 0.0 | 23.6 | 36.7 | 37.1 | 4226.4 |  |
|  | 11/08/2021 | 8.41 | 8.9 | 2.0 | 6.1 | 9.2 | 1.836 | 0.076 | 4.1 | 0.0 | 22.2 | 32.6 | 30.1 | 272.5 |  |
|  | 13/08/2021 | 9.26 | 7.8 | 1.6 | 6.1 | 12.4 | 1.873 | 0.080 | 4.0 | 0.5 | 22.9 | 28.6 | 27.6 | 6.8 |  |
|  | 20/08/2021 | 7.48 | 5.3 | 8.3 | 2.3 | 7.5 | 2.016 | 0.097 | 5.7 | 0.0 | 19.6 | 30.9 | 28.6 | >5,000 |  |

*MC concentrations were determined by duplicate ELISA measurements. For samples exceeding the assay range, serial 10-fold dilutions were applied, and final concentrations were back-calculated by multiplying the measured value of the diluted sample by the dilution factor. Values within the assay range were retained as measured, whereas values exceeding 5.0 μg/L were rounded to one decimal place. Values above 5,000 μg/L were reported using threshold notation as >5,000. ND indicates values below the limit of detection (0.05 μg/L).

| Study site group | Nearby algal observation station | Date | Water temp. (°C) | Chl-a (mg/m³) | Harmful cyanobacteria (cells/mL) | *Microcystis* (cells/mL) |
| --- | --- | --- | --- | --- | --- | --- |
| S1–S2 | Gangjeong-Goryeong Weir | 2021-07-26 | 29.9 | 31.3 | 89,443 | 86,700 |
| S1–S2 | Gangjeong-Goryeong Weir | 2021-08-02 | 30.1 | 20.3 | 41,956 | 32,980 |
| S1–S2 | Gangjeong-Goryeong Weir | 2021-08-09 | 29.4 | 17.6 | 24,476 | 22,520 |
| S1–S2 | Gangjeong-Goryeong Weir | 2021-08-17 | 27.7 | 15.1 | 2,650 | 2,650 |
| S1–S2 | Gangjeong-Goryeong Weir | 2021-08-23 | 27.0 | 12.3 | 5,470 | 5,470 |
| S3–S4 | Dalseong Weir | 2021-07-26 | 31.8 | 10.2 | 8,492 | 7,710 |
| S3–S4 | Dalseong Weir | 2021-08-02 | 31.4 | 47.3 | 74,942 | 67,950 |
| S3–S4 | Dalseong Weir | 2021-08-09 | 29.8 | 9.3 | 3,840 | 3,715 |
| S3–S4 | Dalseong Weir | 2021-08-17 | 27.8 | 14.1 | 4,760 | 4,760 |
| S3–S4 | Dalseong Weir | 2021-08-23 | 26.4 | 17.8 | 32,680 | 32,680 |
| S5–S7 | Hapcheon-Changnyeong Weir | 2021-07-26 | 30.0 | 58.0 | 188,054 | 187,500 |
| S5–S7 | Hapcheon-Changnyeong Weir | 2021-08-02 | 30.3 | 44.6 | 113,360 | 107,400 |
| S5–S7 | Hapcheon-Changnyeong Weir | 2021-08-09 | 29.6 | 8.4 | 1,795 | 1,795 |
| S5–S7 | Hapcheon-Changnyeong Weir | 2021-08-17 | 27.6 | 8.4 | 18,375 | 18,375 |
| S5–S7 | Hapcheon-Changnyeong Weir | 2021-08-23 | 26.4 | 10.6 | 9,620 | 9,620 |
| S8–S9 | Changnyeong-Haman Weir | 2021-07-26 | 29.7 | 50.6 | 16,749 | 14,925 |
| S8–S9 | Changnyeong-Haman Weir | 2021-08-02 | 30.7 | 42.9 | 43,366 | 41,200 |
| S8–S9 | Changnyeong-Haman Weir | 2021-08-09 | 28.7 | 14.1 | 2,317 | 2,260 |
| S8–S9 | Changnyeong-Haman Weir | 2021-08-17 | 27.2 | 28.6 | 40,233 | 40,100 |
| S8–S9 | Changnyeong-Haman Weir | 2021-08-23 | 26.1 | 14.6 | 12,443 | 12,380 |

**Table S5.** Public algal monitoring data from nearby algal observation stations in the Nakdong River during July–August 2021.

Public monitoring data were obtained from nearby algal observation stations in the Water Environment Information System. These data were included to provide regional context for bloom conditions during the study period and were not sample-matched to the present study.

|  | ***R²*** | ***p*** |
| --- | --- | --- |
| **Bacteria** |  |  |
| ASV | 0.039 | 0.003 |
| Phylum | 0.583 | 0.001 |
| Class | 0.527 | 0.001 |
| Order | 0.489 | 0.001 |
| **Microeukaryota** |  |  |
| ASV | 0.173 | 0.001 |
| Phylum | 0.140 | 0.001 |
| Class | 0.151 | 0.001 |
| Order | 0.278 | 0.001 |

**Table S6.** PERMANOVA results assessing differences among cyanobacterial ratio groups (High, Middle, and Low) based on Bray–Curtis dissimilarities at multiple taxonomic levels (ASV, phylum, class, order) for bacterial and microeukaryotic communities. *R²* values represent the proportion of variance explained by group identity, and *p*-values were obtained using 999 permutations.


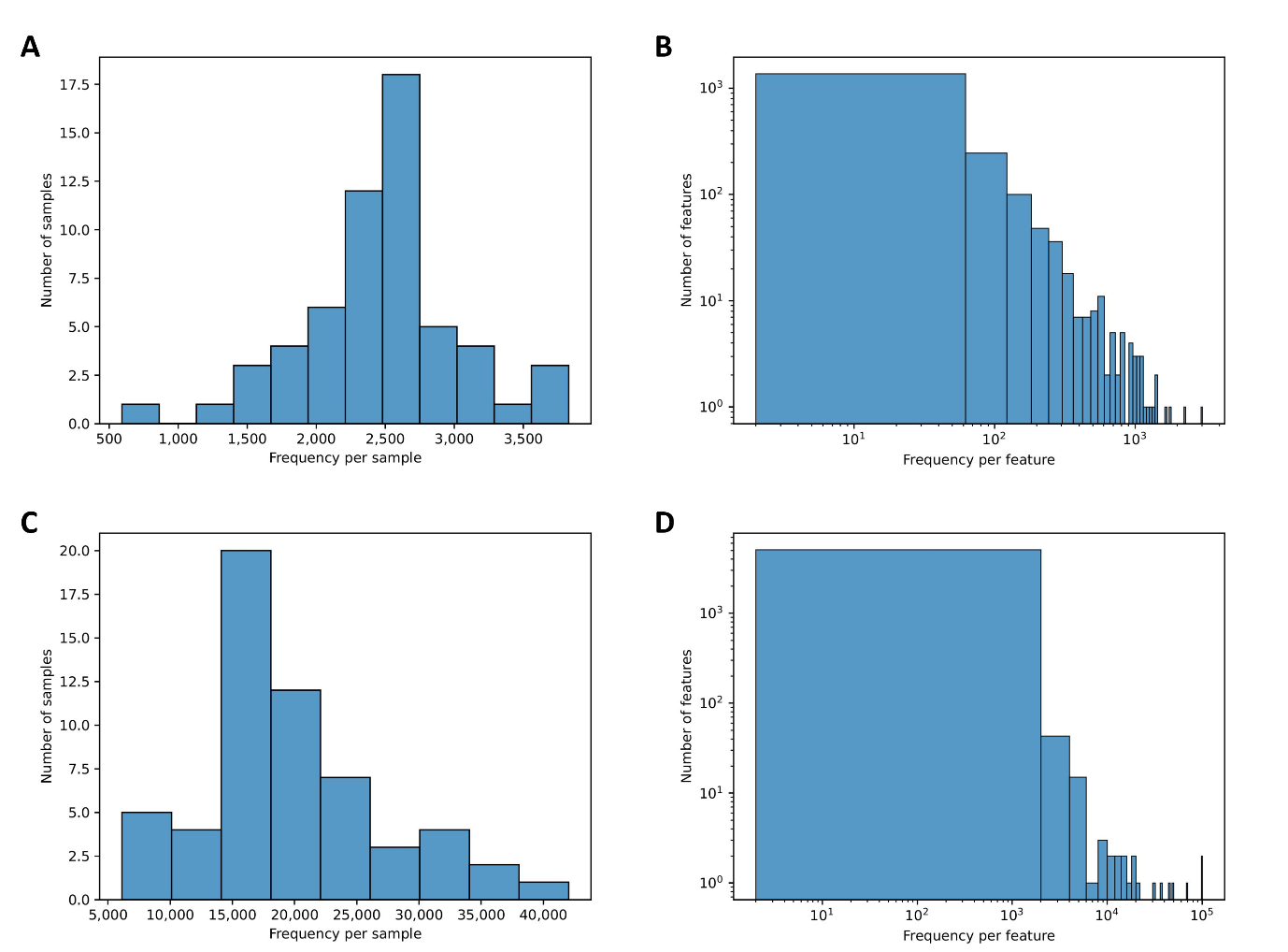


**Figure S1.** Read depth and feature-frequency distributions used to select rarefaction depths. (A–B) Bacterial dataset: per-sample sequencing depth histogram showing a minimum of 593 reads (A) and per-feature frequency distribution (B). (C–D) Microeukaryotic dataset: per-sample sequencing depth histogram showing a minimum of 6,119 reads (C) and per-feature frequency distribution (D). For the bacterial dataset, although the minimum observed read depth was 593, the final rarefaction depth used for downstream analyses was set to 1,137 reads per sample after excluding the lowest-depth sample. The microeukaryotic dataset was rarefied to 6,119 reads per sample.


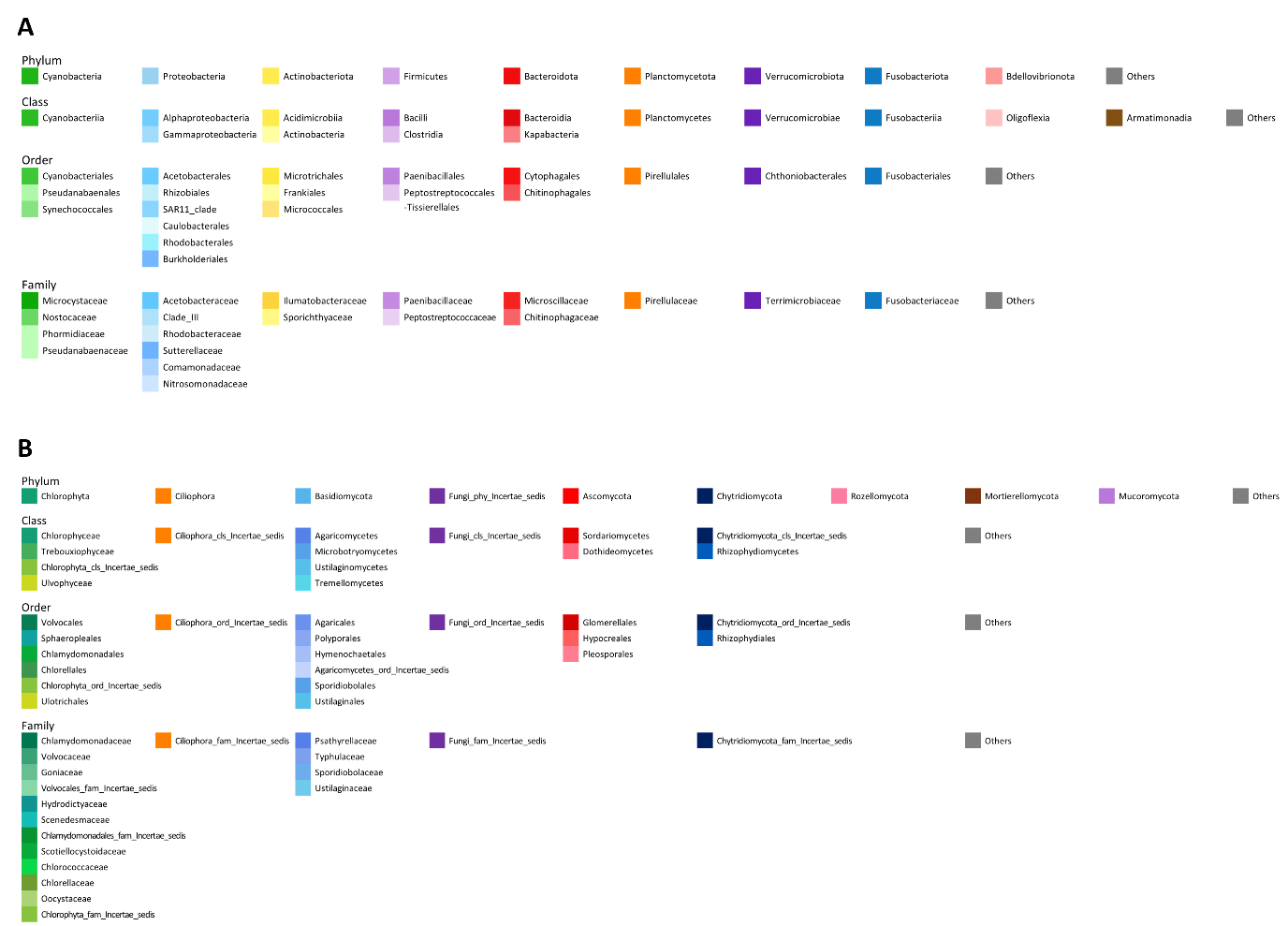


**Figure S2.** Taxonomic color legend for bacterial and microeukaryotic communities shown in Figure 2. (A) Bacterial community and (B) Microeukaryotic community. Color codes correspond to taxa from the phylum to family levels, applied consistently in stacked bar plots and concentric donut pie charts.

**
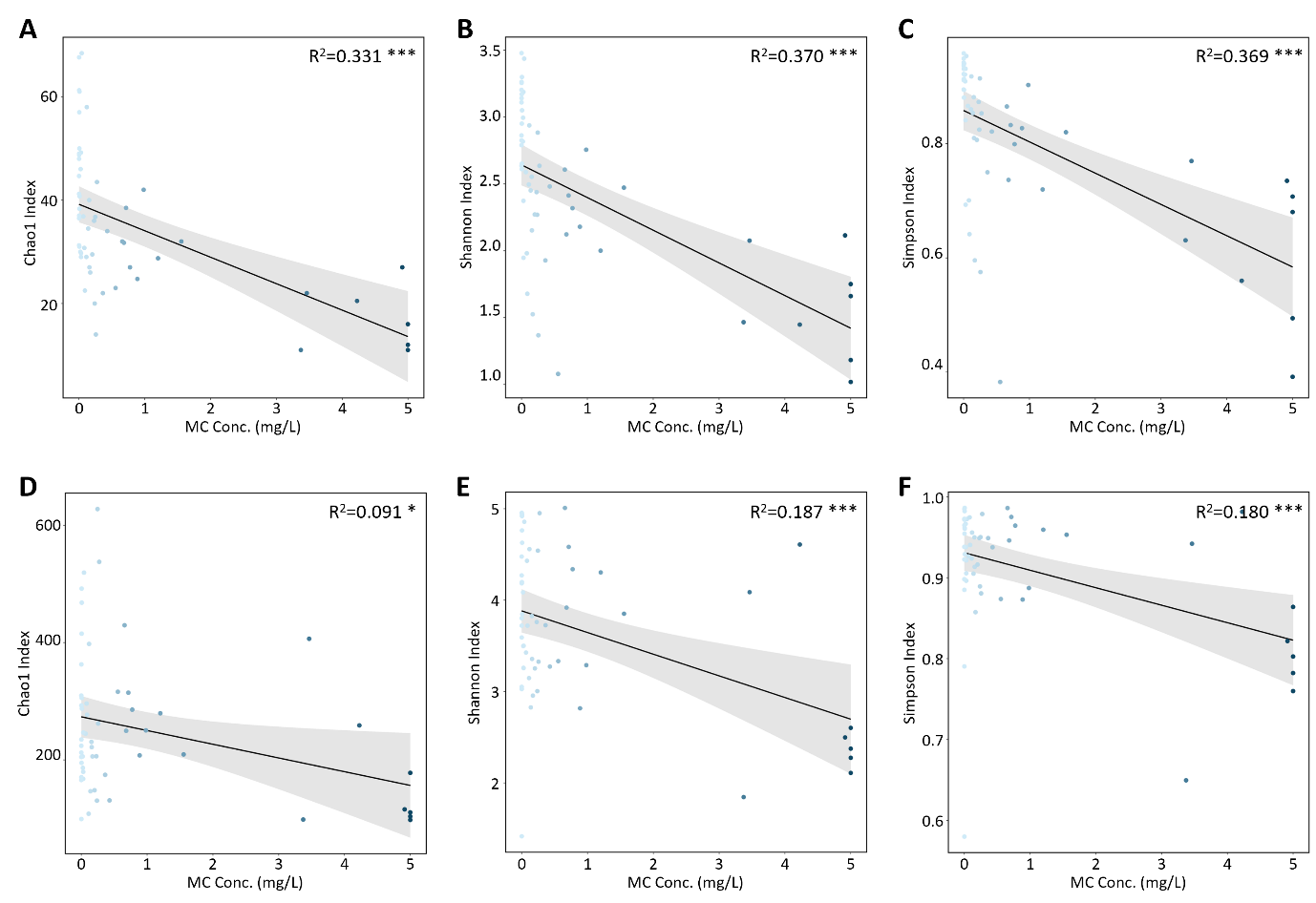
Figure S3.** Linear regression analysis of alpha diversity indices (Chao1, Shannon, Simpson) against microcystin (MC) concentrations. (A–C) Bacterial community and (D–F) Microeukaryotic community. Each panel shows a linear regression model with the coefficient of determination (*R²*). Significance is indicated by *(*p* < 0.05), **(*p* < 0.01), and ***(*p* < 0.001). Points are colored with a gradient from light to dark blue according to MC concentration (μg/L).

**
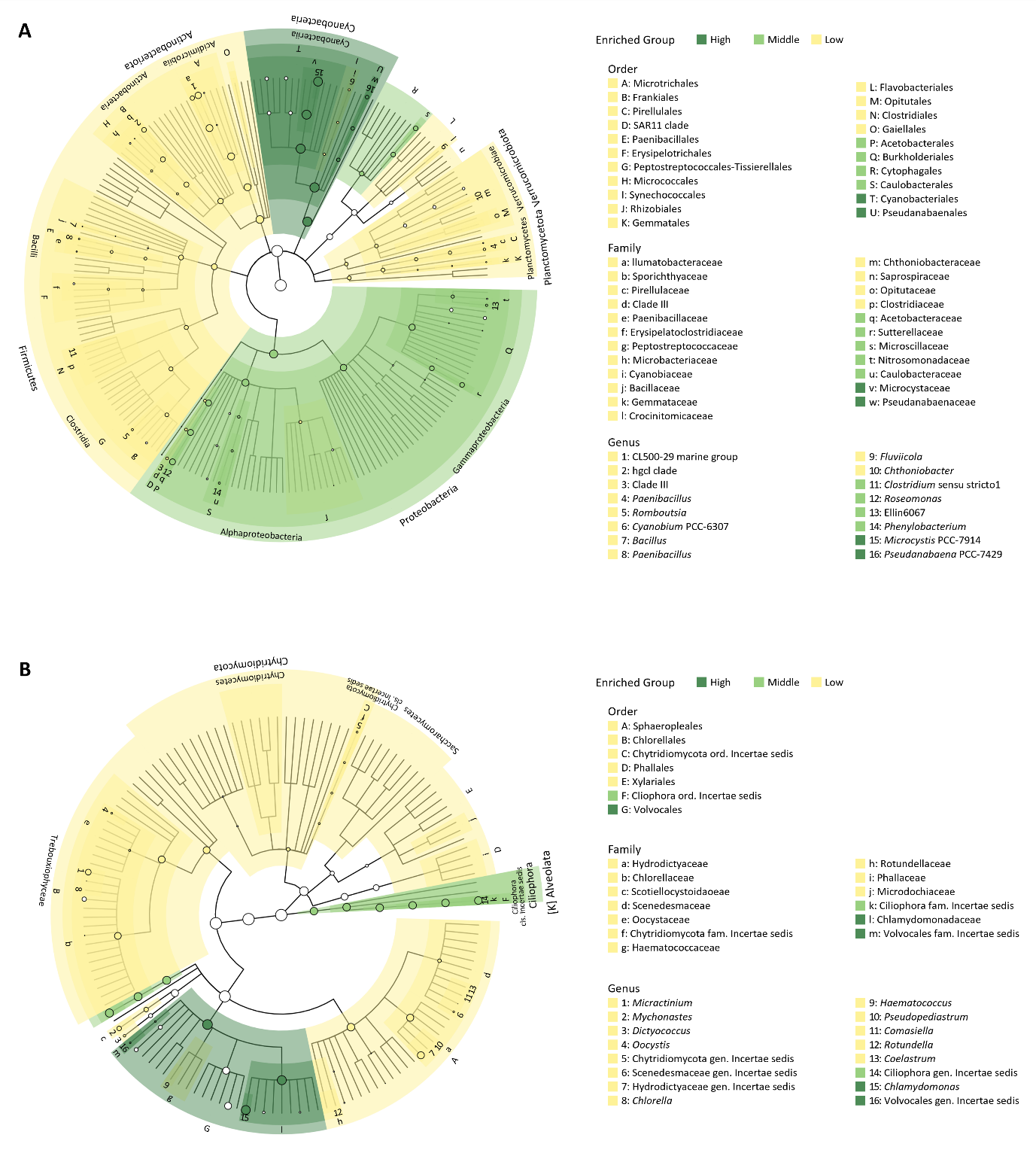
Figure S4.** LEfSe cladograms of bacterial (A) and microeukaryotic (B) communities across cyanobacterial-ratio groups. LEfSe was performed on taxonomically annotated features across hierarchical ranks from phylum to genus. Samples were classified as High (n = 19), Middle (n = 18), and Low (n = 19) according to cyanobacterial ratio. Differentially abundant taxa were identified using a Kruskal–Wallis test (p < 0.01), pairwise Wilcoxon tests (p < 0.05), and an LDA score threshold of 3.0. Taxa enriched in each group are indicated by colored nodes (High, green; Middle, light green; Low, yellow).
